# Prevalence and correlates of cytopenias in HIV-infected adults initiating highly active antiretroviral therapy in Uganda

**DOI:** 10.1186/1471-2334-14-496

**Published:** 2014-09-10

**Authors:** Rachel Kyeyune, Elmar Saathoff, Amara E Ezeamama, Thomas Löscher, Wafaie Fawzi, David Guwatudde

**Affiliations:** Infectious Diseases Institute, Makerere College of Health Sciences, P.O Box 22418, Kampala, Uganda; Center for International Health, Ludwig-Maximillians University, Munich, Germany; Department of Epidemiology and Biostatistics, Makerere School of Public Health, Kampala, Uganda; Division of Infectious Diseases and Tropical Medicine, Medical Center of the University of Munich, Munich, Germany; German Center for Infection Research (DZIF), partner site Munich, Germany; Department of Epidemiology and Biostatistics, the University of Georgia, Georgia, USA; Department of Nutrition, Department of Epidemiology & Department of Global Health and Population, Harvard School of Public Health, Boston, USA

**Keywords:** Hematological abnormalities, HIV, Cytopenia, Uganda

## Abstract

**Background:**

Cytopenias are the most common HIV-associated hematological abnormality. Cytopenias have been associated with several factors including sex, race/ethnicity, geographical location and comorbidities such as tuberculosis, hepatitis B infection, fever and oral candidiasis. Cytopenias become more prevalent as HIV progresses and are often fatal. Data from resource-limited settings about the prevalence and correlates of cytopenia are limited. Therefore we conducted this cross-sectional study to assess the prevalence and correlates of cytopenia among adult AIDS patients at initiation of HAART in Uganda.

**Methods:**

400 HIV-infected subjects who were HAART-naïve or on HAART for ≤ 6 months were enrolled into the Multivitamins, HAART and HIV/AIDS Trial. Anemia was defined according to WHO guidelines as any hemoglobin concentration < 12 g/dl for non-pregnant females and < 13 g/dl for males. Leucopenia and thrombocytopenia were defined using study site laboratory reference ranges for lack of generally accepted definitions for these 2 cell lines as leucopenia if white blood cell count < 2.75 × 10^9^ cells/litre and thrombocytopenia if platelets < 125 × 10^9^ cells/litre for females and < 156 × 10^9^ cells/litre for males. Univariate and bivariate analyses were done to describe the patient population and log-binomial regression was used to quantify the correlates of cytopenia.

**Results:**

Sixty five percent of the 400 subjects had at least one form of cytopenia. Anemia occurred in 47.8%, leucopenia in 24.3%, thrombocytopenia in 8.3%, bicytopenia in 21.9% and only 2 had a pancytopenia. Cytopenia was more prevalent in females (prevalence ratio [PR]:1.33, 95% confidence interval [CI]:1.12-1.59); CD4 count category 50 to <200 (PR: 0.75, 95% CI: 0.64 -0.88) and CD4 count category 200 to <350 (PR: 0.74, 95% CI: 0.59 - 0.92) compared to CD4 count category <50; normal BMI (PR: 0.82, 95% CI:0.68-1.00) and overweight BMI (PR: 0.64, 95% CI:0.50- 0.82) compared to underweight BMI and those with a history or presence of oral candidiasis.

**Conclusions:**

Cytopenias are a frequent complication in HIV-infected adults at initiation of HAART in Uganda. The presence of any cytopenia was associated with female sex, decreasing CD4 count and decreasing body mass index. Prospective studies in resource-limited settings on the trend in HIV-related cytopenias are needed.

## Background

Human Immunodeficiency Virus (HIV) infection is associated with hematological abnormalities, the most common manifestation being characterized by reduction in and impaired function of all blood cell lines: red blood cells (anemia), white blood cells (leucopenia or neutropenia) and platelets (thrombocytopenia) collectively called cytopenias. Neutropenia is defined by abnormally low numbers of neutrophils which normally make up 50-70% of circulating white blood cells. Cytopenias increase in frequency as HIV progresses and are often fatal without intervention [1–4].

Anemia is the most common cytopenia and its prevalence varies with different definitions used and in different settings. A systematic review of studies documenting the prevalence or incidence of anemia in HIV-infected patient populations reported prevalence rates from 1.3% to 95% [5]. In Europe and the United States, anemia occurs in about 35-65% of individuals before or at the start of highly active antiretroviral therapy (HAART) [2, 6] and in Africa and Asia the prevalence ranges from 18% to 77% [7–9]. In Uganda the DART trial reported a prevalence of 0.7% grade 4 anemia at week 4 in patients initiating triple combination antiretroviral therapy (ART) [10], Mugisha et al. found 18.9% anemic HIV-positive subjects in a rural cohort of HIV-infected and HIV negative adults [11] and Mukaya et al. reported 64.6% anemic patients attending the medical emergency ward of Uganda’s national referral hospital, Mulago [12]. The presence of anemia has been associated with faster disease progression and has been shown to independently predict survival [1, 2, 13–16]. The widespread use of highly active antiretroviral therapy (HAART) has led to significant improvements in patient survival and more specifically in improvement of anemia particularly severe anemia irrespective of the use of azidothymidine (AZT) in the regimen [2, 8, 10, 17–20]. However, mild to moderate anemia occurs or persists in some individuals on long-term therapy and gradually impairs the individual’s functional status [2, 8, 21]. This is clinically significant because anemia affects quality of life which in the context of HAART has become an increasingly important treatment goal [22, 23].

Data about the prevalence of leucopenia and thrombocytopenia and factors associated with their presence in HIV-infected adults is limited. Leucopenia occurs in 57 to76% of patients with advanced HIV infection [24–26], neutropenia specifically was found in 44% of HIV-infected women prior to initiating HAART [27] while prevalent thrombocytopenia ranges from 7% to about 21% increasing with the stage of HIV infection [7][28]. Neutropenia may be caused by adverse drug reactions or myelosuppressive drugs [7] and it has been associated with low baseline CD4 count [29]. Thrombocytopenia has been independently associated with chronic active hepatitis B virus infection, male gender, low absolute neutrophil count and low CD4 count [15, 30]. However, a study in India did not find a significant correlation between thrombocytopenia and low CD4 count [7].

Cytopenia in HIV-1 infected adults has been associated with geographical location (anemia and neutropenia were highest in Southern African countries and Haiti compared to the US) [30], race/ethnicity (anemia was more prevalent in African-Americans [1, 31] and neutropenia was higher in non-Hispanics than Hispanics [30]), female sex (anemia and neutropenia were higher in females) [1, 6, 9, 11, 30, 31], age (anemia is more prevalent in older patients compared to younger patients) [6] and laboratory markers such as low mean corpuscular volume (MCV: < 80 femtolitres) [20, 31], CD4 cell count (low CD4 count has been associated with prevalent anemia, neutropenia and thrombocytopenia) [7, 11, 29, 31], high viral load [31], use of AZT [31], low body mass index (BMI) [9, 10] and co-morbid conditions such as tuberculosis [9, 20], hepatitis B (thrombocytopenia was associated with hepatitis B surface antigen) [30], malaria, fever, pneumonia and oral candidiasis [11] .

Data about the prevalence and factors associated with cytopenia in patients initiating HAART has largely been derived from developed countries that may differ from developing countries in patient profile, cytopenia etiology, antiretroviral regimen options and comorbidities [1, 2, 13, 14, 19, 31, 32].

The magnitude of pre-existing cytopenias and associated factors at the time of initiating HAART could impact on ART treatment outcomes. However, the magnitude of this problem has not been adequately described in resource-limited settings. This cross-sectional study is designed to fill these gaps by investigating the prevalence and correlates of cytopenia among adult Ugandan AIDS patients either HAART-naïve at enrollment or on HAART for ≤ 6 months. We hypothesize that this population will have higher prevalences of each cytopenia and worse clinical parameters compared to similar cohorts in developed countries. This analysis was approved by the Infectious Diseases Institute Scientific Review Committee, and the Higher Degrees, Research and Ethics Committee (HDREC) of the Makerere School of Public Health.

## Methods

### Study design, setting and population

In this article we present hematological data which were collected at enrolment into a double blind placebo-controlled micronutrient trial among HIV-positive adults ≥ 18 years in Uganda. The adult HIV prevalence in Uganda is estimated at 7.3% [33]. Between March 2010 and June 2012, 400 HIV-positive adults were enrolled into the Multivitamins, HAART and HIV/AIDS Trial. The inclusion criteria for enrolment into the parent trial included being at least 18 years old at the time of enrolment, HIV-positive and eligible to initiate HAART or have been on HAART for not more than 6 months at the time of enrolment, living within a 20 kilometre radius of the health unit of enrolment, without an intention of migrating out of Kampala for at least 18 months after enrolment and provide informed written consent for participation. To be eligible for initiation of HAART, subjects had to present with either WHO stage 4 or CD4 count less than 200 cells/μl, or WHO stage 3 or CD4 count less than 350 cells/μl. Women who were pregnant and those who were ineligible to initiate HAART at the time of enrolment due to early-stage disease were excluded from the trial. Further details of the design and methods of the trial are described elsewhere [34]. Patients were clinically managed at the Infectious Diseases Institute (IDI), Makerere University College of Health Sciences and this trial was implemented at IDI in collaboration with the Makerere School of Public Health and the Harvard School of Public Health. All data presented here were collected at baseline, before the start of the micronutrient intervention.

At enrollment all subjects had a Complete Blood Count (CBC, Beckman Coulter Act 5 Diff, Miami, Florida, USA), and CD4 cell count (BD FACSCalibur System, Becton Dickinson, San Jose, California, USA) done provided this had not been done within a month prior to enrolment. Blood was collected in an EDTA vacutainer for both tests.

### Measurements

#### Anemia

Anemia was defined according to WHO guidelines as <12 g/dl for non-pregnant women and <13 g/dl for men. Anemia severity was graded as mild: 11–11.9 g/dl for women and 11–12.9 g/dl for men; moderate: 8–10.9 g/dl for both sexes; and < 8 g/dl as severe anemia for both sexes [35].

#### Other cytopenias

There are no generally accepted cut-offs for other cytopenias. A study in India used a cut off of total white blood cells < 4000 cells/μl to define leucopenia and platelet count <150 × 10^3^ cells/μl to define thrombocytopenia in HIV-infected individuals [7]. We used the study site laboratory reference ranges (Makerere University-John Hopkins University, MUJHU Core laboratory) to define leucopenia and thrombocytopenia. The MUJHU Core-laboratory is a CAP-certified laboratory (CAP certificate number 7139001). Other cytopenias were thus defined as: leucopenia if total white blood cell count < 2.75 × 10^9^cells/litre, thrombocytopenia if platelet count <125 × 10^9^ cells/litre for females and <156 × 10^9^ cells/litre for males; bicytopenia if a subject had a combination of any 2 cytopenias and pancytopenia as having all three forms of cytopenia simultaneously.

### Statistical analysis

Univariate analysis was used to describe the subjects’ characteristics. Continuous variables were summarized using means or medians as found appropriate and categorical variables were summarized using frequencies and percentages. Thus prevalences of the various forms of cytopenia are reported as percentages of subjects with cytopenias with the denominator being all subjects in the trial.

We used the log-binomial regression model to investigate the factors associated with cytopenias in general and then anemia, leucopenia and thrombocytopenia were considered as individual outcome variables. Prevalence ratios (PR) and their 95% confidence intervals (95%CI) are reported. The independent factors investigated included demographic variables (e.g. sex, age, marital and employment status, highest level of education attained) and clinical/laboratory characteristics (e.g. CD4 cell count, body mass index, presence or history of oral candidiasis, presence or history of fever, etc.).

All data analysis was done using Stata version 11.2.

## Results

### Patient characteristics

This analysis included the 400 subjects enrolled in the parent trial of which 277(69.3%) were female and overall the mean age of the study population was 36 years (Table 1). The median CD4 cell count was 142 cells/μl and 50% of the subjects had initiated antiretroviral therapy at the time of enrolment. The mean duration on HAART was 2.5 months (±1.6 SD). The mean BMI was 23.8 (SD ±9.2),

**Table 1:** **Characteristics and hematological values of the study population at baseline**

N = 400†	
**Socio-demographic characteristics**	Value
Female, n (%)	277 (69.3)
Age, mean (±SD) years	36 (9.0)
Married	135 (33.8%)
Employed	345 (82.3)
Education level(completed at least primary)*	387 (96.8%)
**Immunological, HAART and nutritional status**	
CD4 cell count, median (IQR) per μl	142 (1–645)
HAART status at recruitment	
On HAART, n (%)	200 (50)
Not yet on HAART, n (%)	200 (50)
Duration on HAART at enrollment, mean (±S.D) months	2.5 ± 1.6
BMI†, mean ± S.D	23.8 ± 9.2
**Hematological parameters, median (IQR)**	
Hemoglobin, g/dl	12.3 (4.9-17.6)
White blood cell count × 10^9^ per litre	3.4 (1.2-9.9)
Platelets × 10^9^ per litre	244 (11–616)
Mean corpuscular volume, femtolitres (fl)	86 (58–126)
Red cell Distribution Width (RDW),%	13.6 (9–25.1)

^†^ Data for BMI were missing for 3 participants due to missing weights at baseline.

*One participant had education level as “unknown”.

S.D = Standard Deviation.

IQR = Inter Quartile Range.

μl = microlitre.

### Prevalence of cytopenias

A total of 260 (65%) had at least one form of cytopenia. Anemia was the most common cytopenia occurring in 192 (47.8%) of the subjects followed by leucopenia occurring in 96 (24.3%), thrombocytopenia in 32 (8.3%), bicytopenia in 88 (21.9%) and only 2 had a pancytopenia (Table 2). Cytopenias were more prevalent in subjects aged between 30 to 35 years and in those with CD4 count less than 50 cells/μl.

**Table 2:** **Distribution of Cytopenias across the study population**

	n	% Any Cytopenia	% Anemia	% Leucopenia	% Thrombocytopenia
All participants	400	65.0	47.8	24.3	8.3
Characteristic					
Gender					
Male	123	56.9	34.1	23.6	14.6
Female	277	68.6	53.8	24.5	5.4
Age					
18 to < 30	98	63.3	45.9	23.5	5.1
30 to < 35	80	70.0	50.0	26.3	11.3
35 to < 41	117	64.1	45.3	24.8	8.5
≥ 41	105	63.8	50.5	22.9	8.6
Education level					
None	12	66.7	33.3	41.7	8.3
Minimum Primary	206	65.5	48.5	21.8	8.7
Minimum Secondary	141	66.7	50.4	25.5	8.5
Tertiary	40	57.5	40.0	27.5	5.0
Unknown†	1	0.0	0.0	0.0	0.0
Employment status					
Unemployed	55	69.1	56.4	20.0	3.6
Employed	345	64.3	46.4	24.9	9.0
Marital status					
Not married	265	64.9	49.1	22.3	7.2
Married	135	65.2	45.2	28.1	10.4
CD4 count cells/μl					
<50	59	84.7	50.8	52.5	15.3
50 to <200	241	62.2	49.0	20.3	6.6
200 to < 350	88	59.1	43.2	15.9	9.1
≥ 350	12	66.7	41.7	25.0	0.0
BMI					
under weight	31	83.9	71.0	12.9	9.7
normal	261	66.7	50.2	24.1	8.8
over weight	105	54.3	34.3	25.7	6.7
Incomplete information*	3	100.0	66.7	100.0	0.0
HAART status at recruitment					
Not using HAART	200	68.0	47.0	26.0	12.0
AZT-based HAART	15	66.7	60.0	6.7	0.0
Non-AZT based HAART	185	61.6	47.6	23.8	4.9
Presence/history of oral candidiasis					
No	305	62.0	44.9	23.3	7.5
Yes	95	74.7	56.8	27.4	10.5
Presence/history of fever					
No	195	63.6	44.6	26.7	8.2
Yes	205	66.3	50.7	22.0	8.3

^†^ One participant had education level as “unknown“

*Three participants did not have records for baseline weight therefore BMI could not be computed for these thus categorized as “Incomplete information”.

### Factors associated with cytopenias

In this analysis the factors found to be associated with any cytopenia were female sex, CD4 cell count and body mass index (Table 3). The prevalence ratio of any cytopenia among females compared to males was 1.21 (95% confidence interval [CI]: 1.01-1.43). The prevalence ratio (PR) of having any form of cytopenia decreased with increasing CD4 count; for CD4 count category 50 to < 200 cells/μl PR = 0.73 [95% CI: 0.63- 0.85, p< 0.001], and for CD4 count category 200 to <350 PR = 0.70 [95% CI: 0.57-0.86]. Similarly the prevalence ratio of having any form of cytopenia decreased with increasing BMI; for normal compared to underweight BMI PR = 0.79 [95%CI: 0.67-0.95] and overweight compared to underweight BMI PR = 0.65 [95%CI: 0.51-0.82, p < 0.001]. Anemia was associated with female sex, decreasing BMI and history or presence of oral candidiasis (Table 4). The prevalence ratio of having anemia among females compared to males was 2.24 [95% CI: 1.44-3.49,p<0.001]. The prevalence ratio of having anemia decreased with increasing CD4 count and increasing BMI although the difference by CD4 category was not statistically significant. With respect to BMI the PR for having anemia with a normal BMI was 0.41[95% CI: 0.18-0.93,p=0.033] and for overweight BMI PR: 0.21[95%CI: 0.09-0.51,p=0.001]. Thrombocytopenia was associated with sex, HAART status at the time of enrolment and CD4 count category 50 to < 200 cells/μl (Table 5). The PR of having thrombocytopenia among females compared to males was 0.32 [95% CI: 0.15-0.68, p = 0.003]. The PR of having thrombocytopenia among those on HAART compared to those who were not yet on HAART at enrolment was 0.32 [95% CI: 0.14- 0.75, p = 0.008]. The only factor associated with the presence of leucopenia was CD4 count (Table 6); the PR for CD4 count category 50 to <200 cells/μl compared to CD4 count category <50 cells/μl was 0.23 [95%CI: 0.13-0.42, p <0.001] and PR for CD4 count 200 to <350 cells/μl compared to CD4 count category <50 cells/μl was 0.17 [95%CI: 0.08-0.37, p <0.001].

**Table 3:** **Factors associated with the presence of any cytopenia**

Results of uni- and multivariable log-binomial regression models				
Covariate	Univariable (n = 400)		Multivariable‡ (n = 397*)	
	PR (95% CI)	p value	PR (95% CI)	p value
Sex				
Male	1		1	
Female	1.21 (1.01 to 1.43)	0.035	1.33 (1.12 to 1.59)	0.002
CD4 count cells/μl				
<50	1		1	
50 to < 200	0.73 (0.63 to 0.85)	<˂0.001	0.75 (0.64 to 0.88)	<0.001
200 to < 350	0.70 (0.57 to 0.86)	0.001	0.74 (0.59 to 0.92)	0.007
≥ 350	0.79 (0.52 to 1.19)	0.256	0.77 (0.50 to 1.19)	0.245
BMI				
under weight	1		1	
normal	0.79 (0.67 to 0.95)	0.011	0.82 (0.68 to 1.00)	0.045
over weight	0.65 (0.51 to 0.82)	<0.001	0.64 (0.50 to 0.82)	<0.001
Incomplete information*	-	-	-	-
Presence/history of oral candidiasis				
No	1			
Yes	1.21 (1.04 to 1.40)	0.012		
HAART status at recruitment				
Not using HAART	1			
AZT-based HAART	0.98 (0.68 to 1.42)	0.917		
Non-AZT based HAART	0.91 (0.78 to 1.05)	0.193		
Age, years				
18 to < 30	1			
30 to < 35	1.11 (0.90 to 1.36)	0.341		
35 to < 41	1.01 (0.83 to 1.24)	0.899		
≥ 41	1.01 (0.82 to 1.24)	0.936		
Presence/history of fever				
No	1			
Yes	1.04 (0.90 to 1.21)	0.565		

^‡^Multivariable model only includes the variables where data are shown because all others were lacking significance.

*Three participants with missing BMI could not be included in the log-binomial regression model because the outcome did not vary, thus n = 397 for multivariable analysis and for BMI in univariable analysis.

**Table 4:** **Factors associated with the presence of anemia**

Results of uni- and multivariable log-binomial regression models				
Covariate	Univariable (n = 400)		Multivariable‡ (n = 397*)	
	PR (95% CI)	p value	PR (95% CI)	p value
Sex				
Male	1		1	
Female	2.24 (1.44 to 3.49)	<0.001	2.97 (1.85 to 4.77)	<0.001
BMI				
under weight	1		1	
normal	0.41 (0.18 to 0.93)	0.033	0.43 (0.19 to 0.99)	0.048
over weight	0.21 (0.09 to 0.51)	0.001	0.17 (0.07 to 0.41)	<0.001
Incomplete information*	0.82 (0.07 to 10.20)	0.876	1.69 (0.13 to 21.61)	0.686
CD4 count cells/μl				
<50	1			
50 to <200	0.93 (0.52 to 1.64)	0.795		
200 to <350	0.73 (0.38 to 1.42)	0.361		
≥ 350	0.69 (0.20 to 2.42)	0.563		
Age, years				
18 to < 30	1			
30 to < 35	1.18 (0.65 to 2.13)	0.588		
35 to < 41	0.98 (0.57 to 1.67)	0.928		
≥ 41	1.20 (0.69 to 2.08)	0.516		
HAART status at recruitment				
Not using HAART	1			
AZT-based HAART	1.69 (0.58 to 4.93)	0.336		
Non-AZT based HAART	1.02 (0.69 to 1.53)	0.911		
Education level				
None	1			
Minimum Primary	1.89 (0.55 to 6.46)	0.312		
Minimum Secondary	2.03 (0.58 to 7.04)	0.265		
Tertiary	1.33 (0.34 to 5.18)	0.678		
Unknown†	-	-		
Employment status				
Unemployed	1			
Employed	0.67 (0.38 to 1.19)	0.170		
Marital status				
Not married	1			
Married	0.86 (0.56 to 1.30)	0.464		
Presence/history of oral candidiasis				
No	1			
Yes	1.62 (1.01 to 2.57)	0.043		
Presence/history of fever				
No	1			
Yes	1.28 (0.86 to 1.89)	0.221		

^‡^Multivariable model only includes variables for which data are shown below because all others were lacking significance.

^†^One participant with unknown education level was excluded from analysis because the outcome did not vary in this stratum. *Three subjects with missing BMI could not be not included in the log-binomial regression model because the outcome did not vary, thus n=397 for the multivariable analysis and for the BMI univariable analysis.

**Table 5:** **Factors associated with the presence of thrombocytopenia**

Results of uni- and multivariable log-binomial regression models				
Covariate	Univariable (n = 400)		Multivariable‡ (n = 397*)	
	PR (95% CI)	p value	PR (95% CI)	p value
Sex				
Male	1		1	
Female	0.33 (0.16 to 0.69)	0.003	0.32 (0.15 to 0.68)	0.003
HAART status at recruitment^§^				
Not on HAART	1		1	
On HAART	0.35 (0.16 to 0.76)	0.009	0.32 (0.14 to 0.75)	0.008
CD4 count cells/μl				
<50	1			
50 to < 200	0.40 (0.17 to 0.95)	0.037		
≥ 200**	0.48 (0.18 to 1.33)	0.159		
BMI				
under weight	1			
normal	0.90 (0.25 to 3.20)	0.873		
over weight	0.67 (0.16 to 2.75)	0.575		
Incomplete information*	-	-		
Age, years				
18 to < 30	1			
30 to < 35	2.36 (0.76 to 7.34)	0.139		
35 to < 41	1.74 (0.57 to 5.27)	0.328		
≥ 41	1.74 (0.56 to 5.40)	0.335		
Education level				
None	1			
Minimum Primary	1.05 (0.13 to 8.63)	0.961		
Minimum Secondary	1.02 (0.12 to 8.62)	0.983		
Tertiary	0.58 (0.05 to 7.00)	0.667		
Don’t know	-	-		
Employment status				
Unemployed	1			
Employed	2.62 (0.61 to 11.3)	0.196		
Marital status				
Not married	1			
Married	1.50 (0.73 to 3.09)	0.274		
Presence/history of oral candidiasis				
No	1			
Yes	1.44 (0.66 to 3.15)	0.358		
Presence/history of fever				
No	1			
Yes	1.01 (0.50 to 2.06)	0.975		

^‡^Multivariable model only includes variables for which data are shown below because all others were lacking significance.

§All participants on HAART were categorized into one group for this analysis because none of the 15 patients on AZT had thrombocytopenia.

AZT = azidothymidine or zidovudine.

*Three participants with missing BMI could not be included in the log-binomial regression model because the outcome did not vary in this group.

**CD4 categories 200 to <350 and ≥ 350 were combined for this analysis because none of the participants with CD4 count ≥ 350 had thrombocytopenia.

**Table 6:** **Factors associated with the presence of leucopenia**

Results of uni- and multivariable log-binomial regression models				
Covariate	Univariable (n = 400)		Multivariable‡ (n = 397*)	
	PR (95% CI)	p value	PR (95% CI)	p value
CD4 count cells/μl				
<50	1		1	
50 to <200	0.23 (0.13 to 0.42)	<0.001	0.23 (0.13 to 0.42)	<0.001
200 to <350	0.17 (0.08 to 0.37)	<0.001	0.17 (0.08 to 0.37)	<0.001
≥ 350	0.30 (0.07 to 1.22)	0.094	0.30 (0.07 to 1.22)	0.094
Sex				
Male	1			
Female	1.05 (0.64 to 1.74)	0.834		
Age, years				
18 to < 30	1			
30 to < 35	1.16 (0.59 to 2.30)	0.669		
35 to < 41	1.07 (0.57 to 2.01)	0.822		
≥ 41	0.97 (0.50 to 1.86)	0.918		
BMI*				
under weight	1			
normal	2.15 (0.72 to 6.37)	0.168		
over weight	2.34 (0.75 to 7.29)	0.144		
Incomplete information*	-	-		
HAART status at recruitment				
Not using HAART	1			
AZT-based HAART	0.20 (0.03 to 1.58)	0.128		
Non-AZT based HAART	0.89 (0.56 to 1.41)	0.616		
Education level				
None	1			
Minimum Primary	0.39 (0.12 to 1.29)	0.124		
Minimum Secondary	0.48 (0.14 to 1.61)	0.234		
Tertiary	0.53 (0.14 to 2.03)	0.355		
Don’t know^†^	-	-		
Employment status				
Unemployed	1			
Employed	1.33 (0.66 to 2.69)	0.430		
Marital status				
Not married	1			
Married	1.37 (0.85 to 2.20)	0.195		
Presence/history of oral candidiasis				
No	1			
Yes	1.24 (0.74 to 2.10)	0.417		
Presence/history of fever				
No	1			
Yes	0.77 (0.49 to 1.22)	0.272		

^‡^Multivariable model only includes variables for which data are shown below because all others were lacking significance.

*Three participants with missing BMI could not be included in the log-binomial regression model because the outcome did not vary in this group.

^†^One participant with unknown education level was excluded from analysis because the outcome did not vary in this stratum.

## Discussion

Hematological abnormalities in HIV infected individuals such as cytopenia and in particular anemia have been shown to predict disease progression and mortality [2, 13, 15, 36–39]. The causative role of HIV *in vivo* in altering the bone marrow microenvironment thus inhibiting hematopoiesis and directly resulting in cytopenia is uncertain [40–44]. However, cytopenias occur more frequently with advanced HIV or as viral replication persists and patients may present with multiple cytopenias [3, 4, 45]. We evaluated the types, frequencies and correlates of cytopenia in HIV-infected adults at initiation of HAART in an urban cohort in Uganda. A higher prevalence of all 3 forms of cytopenia was found in this population compared to two other studies in HIV-infected individuals prior to initiation of HAART; one in Korea and another, a multicenter study. The prevalence of anemia was 3% in the Korean study and 11.9% in the multicenter study; neutropenia occurred in 10% and 14.3% and thrombocytopenia in 2.4% and 7.2% respectively [30, 32]. These differences could be due to different cut-off values used to define the cytopenias particularly anemia. Furthermore patients with severe forms of anemia for instance were excluded from both studies and this could have under-estimated the true prevalence. Female sex, increasing immune suppression (measured by CD4 cell count) and decreasing body mass index (BMI) were independent predictors of having a cytopenia in this study. This is consistent with other studies that have found females to be more likely to have anemia (as a result of additional demands during pregnancy and menstrual loss) [9, 11, 30] and to have a higher odds of neutropenia than males [30]. This study also confirms the findings of other studies that have shown an association between low CD4 counts and the presence or development of anemia [6, 31], neutropenia [29, 39] and thrombocytopenia [39]. Furthermore, the highest rates of cytopenia occurred in patients with advanced HIV (CD4 count <200 cell/μl) which is consistent with other studies in developing countries [7, 9, 15, 29]. Out of 191 subjects with anemia in our study, 77% (n = 148) had CD4 count <200. Similarly out of 97 subjects with leucopenia, 82% (n = 80) had CD4 < 200 and of the 33 subjects with thrombocytopenia, 75% (n = 25) had CD4 count <200. However, these differences by CD4 category <200 or ≥ 200 were not statistically significant. The association of low CD4 cell count with anemia and leucopenia may be due to the dysregulatory effect of HIV on the function of early hematopoietic progenitor cells through the viral accessory protein Negative factor (Nef) [3]. Anemia was the most prevalent cytopenia and female sex and decreasing BMI were the sole predictors of having anemia in this study. The prevalence of anemia was considerably higher compared to a rural Ugandan cohort which found 18.9% anemic HIV-positive individuals even though lower hemoglobin cut-offs were used in the rural cohort and the CD4 cell counts were comparable in both cohorts [11]. This could be due to differences in modifiable factors like types of diet, alcohol consumption, cigarette smoking and so on which may vary between urban and rural populations however, we could not confirm this. Subbaraman et al. attributed the association between low BMI and anemia to nutrient deficiencies and chronic malnutrition which is prevalent in resource-limited settings [9]. A study in Brazil found a lower prevalence of anemia (37.7%) compared to our study and this was partly attributed to improvements in the country’s social and nutritional conditions over recent decades [15]. The etiology of anemia in HIV-infection is multifactorial and has been attributed to infiltrative conditions of the bone marrow (e.g. neoplasms, drugs or infections including HIV itself) [16, 46], hemolytic causes (e.g. red blood cell autoantibodies [47], drugs [48]), decreased production of or response to endogenous erythropoietin [49] and nutritional deficiencies [50]. Without intervention, anemia can lead to significant symptoms like fatigue, breathlessness, difficulty in concentration and other effects on functionality and quality of life therefore treatment of anemia in HIV should be aimed at correcting the underlying cause [16]. Regarding leucopenia, neutropenia is the most clinically relevant subtype because it is a good indicator of the risk of infection. In this study, decreasing CD4 count was the sole predictor of having leucopenia. The presence of neutropenia in HIV infection exacerbates the susceptibility to infections such as bacteremia, meningitis but particularly to invasive fungal infections [51]. The etiology of neutropenia is multifactorial and correction of potentially reversible causes is critical to management of HIV-associated neutropenia [51]. Thrombocytopenia was less commonly observed than anemia and leucopenia in our study and this is comparable to findings in other studies [30, 32]. Being female PR 0.32 [95% CI: 0.15-0.68, p = 0.003] and being on HAART at the time of enrolment PR 0.32 [95% CI: 0.14-0.75, p = 0.008] were associated with decreased odds of having thrombocytopenia. Thrombocytopenia is caused mainly by inadequate platelet production or immune-mediated platelet destruction [16]. Of the 400 subjects, 200 (50%) were already on HAART at the time of enrolment and 93% of these were on an AZT-based regimen. AZT has been shown to be the treatment of choice for HIV-related thrombocytopenia [52]and this could explain the relatively low prevalence of this cytopenia found in our study. However, because abnormal bleeding in these patients is not very common (i.e. when platelet counts are > 30,000 × 10^9^/L) [53], treatment of mild to moderate HIV-related thrombocytopenia may not be necessary [16, 53, 54].

The main limitation of this analysis is its cross-sectional design which makes determination of temporal relationships between cytopenias and associated factors difficult. Furthermore we did not assess the various causes of cytopenia and specifically anemia in this population.

## Conclusion

In conclusion, this study provides an opportunity to compare the prevalence estimates and correlates of all forms of cytopenia in HIV-infected adults at initiation of HAART in Uganda. The prevalence of anemia was the highest and meets WHO criteria for a severe public health problem. Female sex, decreasing CD4 count and decreasing BMI were the main factors associated with having cytopenia. Prospective studies in resource-limited settings on the trend in hematological parameters are needed. Hematological parameters are potential markers for disease progression and treatment outcomes in HAART-treated individuals in these settings.

## Abbreviations

HIV: Human immunodeficiency virus
HAART: Highly active antiretroviral therapy
AIDS: Acquired immunodeficiency deficiency syndrome
BMI: Body mass index
AZT: Azidothymidine.

## References

[1] Sullivan PS, Hanson DL, Chu SY, Jones JL, Ward JW, The Adult/Adolescent Spectrum of Disease Group (1998). Epidemiology of anemia in human immunodeficiency virus (HIV)-infected persons: results from the multistate adult and adolescent spectrum of HIV disease surveillance project. Blood.

[2] Mocroft A, Kirk O, Barton SE, Dietrich M, Proenca R, Colebunders R, Pradier C, d Arminio Monforte A, Ledergerber B, Lundgren JD (1999). Anemia is an independent predictive marker for clinical prognosis in HIV infected patients from across Europe. EuroSIDA study group. AIDS.

[3] Kirchhoff F, Silvestri G (2008). Is Nef the elusive cause of HIV-associated hematopoietic dysfunction?. J Clin Investig.

[4] Zon LI, Arkin C, Groopman JE (1987). Haematologic manifestations of the human immune deficiency virus (HIV). Br J Haematol.

[5] Belperio PS, Rhew DC (2004). Prevalence and outcomes of anemia in individuals with human immunodeficiency virus: a systematic review of the literature. Am J Med.

[6] The Antiretroviral Therapy Cohort Collaboration (2007). Prognostic importance of anaemia in HIV type-1-infected patients starting antiretroviral therapy: collaborative analysis of prospective cohort studies. Antivir Ther.

[7] Dikshit B, Wanchu A, Sachdeva R, Sharma A, Das R (2009). Profile of hematological abnormalities of Indian HIV infected individuals. BMC Blood Disorders.

[8] Johannessen A, Naman E, Gundersen SG, Bruun JN (2011). Antiretroviral treatment reverses HIV-associated anemia in rural Tanzania.

[9] Subbaraman R, Devaleenal B, Selvamuthu P, Yepthomi T, Solomon SS, Mayer KH, Kumarasamy N (2009). Factors associated with anaemia in HIV-infected individuals in southern India. Int J STD AIDS.

[10] Ssali F, Stöhr W, Munderi P, Reid A, Walker AS, Gibb DM, Mugyenyi P, Kityo C, Grosskurth H, Hakim J, Byakwaga H, Katabira E, Darbyshire JH, Gilks CF, DART Trial Team (2006). Prevalence, incidence and predictors of severe anemia with zidovudine-containing regimens in Afircan adults with HIV infection within the DART trial. Antivir Ther.

[11] Mugisha JO, Shafer LA, Der Paal LV, Mayanja BN, Eotu H, Hughes P, Whitworth JAG, Grosskurth H (2008). Anaemia in a rural Ugandan HIV cohort: prevalence at enrolment, incidence, diagnosis and associated factors. Trop Med Int Health.

[12] Mukaya JE, Ddungu H, Ssali F, O’Shea T, Crowther MA (2009). Prevalence and morphological types of anaemia and hookworm infestation in the medical emergency ward, Mulago Hospital, Uganda. S Afr Med J.

[13] Moore RD, Keruly JC, Chaisson RE (1998). Anemia and survival in HIV infection. J Acquir Immune Defic Syndr.

[14] Turner BJ, Markson L, Taroni F (1996). Estimation of survival after AIDS diagnosis: CD4 T lymphocyte count versus clinical severity. J Clin Epidemiol.

[15] De Santis GC, Brunetta DM, Vilar FC, Brandão RA, de Albernaz Muniz RZ, de Lima GMN, Amorelli-Chacel ME, Covas DT, Machado AA (2011). Hematological abnormalities in HIV-infected patients. Int J Infect Dis.

[16] Coyle TE (1997). Hematologic complications of human immunodeficiency virus infection and the acquired immunodeficiency syndrome. Med Clin North Am.

[17] The Antiretroviral Therapy Cohort Collaboration (2008). Life expectancy of individuals on combination antiretroviral therapy in high-income countries: a collaborative analysis of 14 cohort studies. Lancet.

[18] Semba RD, Shah N, Klein RS, Mayer KH, Schuman P, Gardner LI, Vlahov D, HER (Human Immunodeficiency Virus Epidemiology Research) Study Group (2001). Highly active antiretroviral therapy associated with improved anemia among HIV-infected women. AIDS PATIENT CARE and STDs.

[19] Moore RD, Forney D (2002). Anemia in HIV-infected patients receiving highly active antiretroviral therapy. J Acquir Immune Defic Syndr.

[20] Kiragga AN, Castelnuovo B, Nakanjako D, Manabe YC: Baseline severe anemia should not preclude use of zidovudine in antiretroviral-eligible patients in resource-limited settings. J Int AIDS Soc. 2010, 13 (42):10.1186/1758-2652-13-42 PMC2991285 21047391

[21] Moore RD (1999). Human immunodeficiency virus infection, anemia, and survival. Clin Infect Dis.

[22] Volberding P (2002). The impact of anemia on quality of life in human immunodeficiency virus—infected patients. J Infect Dis.

[23] Volberding P, for The Anemia in HIV Working Group: Consensus Statement (2000). Anemia in HIV infection—current trends, treatment options, and practice strategies. Clin Ther.

[24] Evans RH, Scadden DT (2000). Haematological aspects of HIV infection. Baillieres Best Pract Res Clin Haematol.

[25] Frontiera M, Myers AM (1987). Peripheral blood and bone marrow abnormalities in the acquired immunodeficiency syndrome. West J Med.

[26] Castella A, Croxson TS, Mildvan D, Witt DH, Zalusky R (1985). The bone marrow in AIDS. A histologic, hematologic and microbiologic study. Am J Clin Pathol.

[27] Levine AM, Karim R, Mack W, Gravink DJ, Anastos K, Young M, Cohen M, Newman M, Augenbraun M, Gange S, Watts DH (2006). Neutropenia in human immunodeficiency virus infection: data from the Women’s interagency HIV study. Arch Intern Med.

[28] Sloand EM, Klein HG, Banks SM, Vareldzis B, Merritt S, Pierce P (1992). Epidemiology of thrombocytopenia in HIV. Eur J Haematol.

[29] Toure S, Gabillard D, Inwoley A, Seyler C, Gourvellec G, Anglaret X (2006). Incidence of neutropenia in HIV-infected African adults receiving co-trimoxazole prophylaxis: a 6-year cohort study in Abidjan, Côte d’Ivoire. Trans R Soc Trop Med Hyg.

[30] Firnhaber C, Smeaton L, Saukila N, Flanigan T, Gangakhedkar R, Kumwenda J, La Rosa A, Kumarasamy N, De Gruttola V, Hakim JG, Campbell TB (2010). Comparisons of anemia, thrombocytopenia, and neutropenia at initiation of HIV antiretroviral therapy in Africa, Asia, and the Americas. Int J Infect Dis.

[31] Levine AM, Berhane K, Masri-Lavine L, Sanchez ML, Young M, Augenbraun M, Cohen M, Anastos K, Newman M, Gange SJ, Watts H (2001). Prevalence and correlates of anemia in a large cohort of HIV-infected women: Women’s interagency HIV study. J Acquir Immune Defic Syndr.

[32] Choi SY, Kim I, Kim NJ, Lee S, Choi Y, Bae J, Kwon JH, Choe PG, Park WB, Yoon S, Park S, Kim BK, Oh M (2011). Hematological manifestations of human immunodeficiency virus infection and the effect of highly active anti-retroviral therapy on cytopenia. Korean Journal of Hematology.

[33] Uganda Ministry of Health and ICF International (2012). Uganda AIDS Indicator Survey: Key Findings.

[34] Guwatudde D, Ezeamama AE, Bagenda D, Kyeyune R, Wabwire-Mangen F, Wamani H, Mugusi F, Spiegelman D, Wang M, Manabe YC, Fawzi WW (2012). Multivitamin supplementation in HIV infected adults initiating antiretroviral therapy in Uganda: the protocol for a randomized double blinded placebo controlled efficacy trial.

[35] World Health Organisation (2011). Haemoglobin Concentrations for the Diagnosis of Anaemia and Assessment of Severity. Department of Nutrition for Health and Development Vitamin and Mineral Nutrition Information System.

[36] O’Brien ME, Kupka R, Msamanga GI, Saathoff E, Hunter DJ, Fawzi WW (2005). Anemia is an independent predictor of mortality and immunologic progression of disease among women with HIV in Tanzania. J Acquir Immune Defic Syndr.

[37] Lundgren JD, Mocroft A (2003). Anemia and survival in human immunodeficiency virus. Clin Infect Dis.

[38] Obirikorang C, Yeboah FA (2009). Blood hemoglobin measurement as a predicitve indicator for HIV/AIDS progression in resource-limited setting.

[39] Sullivan PS, Hanson DL, Chu SY, Jones JL, Ciesielski CA, the Adult/Adolescent Spectrum of Disease Group (1997). Surveillance for thrombocytopenia in persons infected with HIV: results from the multistate adult and adolescent spectrum of disease project. J Acquir Immune Defic Syndr.

[40] Koka PS, Reddy ST (2004). Cytopenias in HIV infection: mechanisms and alleviation of hematopoietic inhibition. Curr HIV Res.

[41] Morgan J, Hanley MB, Moreno MB, Wieder E, McCune JM (1998). Human immunodeficiency virus-1 infection interrupts thymopoiesis and multilineage hematopoiesis in vivo. Blood.

[42] Koka PS, Fraser JK, Bryson Y, Bristol GC, Aldrovandi GM, Daar ES, Zack JA (1998). Human immunodeficiency virus inhibits multilineage hematopoiesis in vivo. J Virol.

[43] Koka PS, Jamieson BD, Brooks DG, Zack JA (1999). Human immunodeficiency virus type 1-induced hematopoietic inhibition is independent of productive infection of progenitor cells in vivo. J Virol.

[44] Moses A, Nelson J, Bagby GC (1998). The influence of human immunodeficiency virus-1 on hematopoiesis. Blood.

[45] Spivak JL, Selonick SE, Quinn TC (1983). Acquired immune deficiency syndrome and pancytopenia. JAMA.

[46] Hambleton J (1996). Hematologic complications of HIV infection. Oncology (Williston Park).

[47] McGinniss MH, Macher AM, Rook AH, Alter HJ (1986). Red cell autoantibodies in patients with acquired immune deficiency syndrome. Transfusion.

[48] Soriano V, Sulkowski M, Bergin C, Hatzakis A, Cacoub P, Katlama C, Cargnel A, Mauss S, Dieterich D, Moreno S, Ferrari C, Poynard T, Rockstroh J (2002). Care of patients with chronic hepatitis C and HIV co-infection: recommendations from the HIV-HCV International Panel. AIDS.

[49] Spivak JL, Barnes DC, Fuchs E, Quinn TC (1989). Serum Immunoreactive Erythropoietin in HIV-Infected Patients. JAMA.

[50] Gregory RH, Phillip DS, McDonald KH, Cecil HF, Scott K, Ernest EL, Clifford L, Anthony SF (1989). Vitamin B12 malabsorption in patients with acquired immunodeficiency syndrome. Arch Intern Med.

[51] Friel TJ, Scadden DT (2013). Hematological Manifestations of HIV Infection: Neutropenia. UpToDate.

[52] Oksenhendler E, Seligmann M (1990). HIV-related thrombocytopenia. Immunodefic Rev.

[53] Landonio G, Nosari A, Spinelli F, Vigorelli R, Caggese L, Schlacht I (1992). HIV-related thrombocytopenia: four different clinical subsets. Haematologica.

[54] Rossi E, Damasio E, Terragna A, Mazzarello G, Spriano M, Anselmo M (1991). HIV-related thrombocytopenia: a therapeutic update. Haematologica.

[CR55] The pre-publication history for this paper can be accessed here:http://www.biomedcentral.com/1471-2334/14/496/prepub

